# Retracted: Technique of Intravesical Laparoscopy for Ureteric Reimplantation to Treat VUR

**DOI:** 10.1155/2016/9495202

**Published:** 2016-02-01

**Authors:** Advances in Urology

The article titled “Technique of Intravesical Laparoscopy for Ureteric Reimplantation to Treat VUR” [1] has been retracted as it was found to be substantially similar to a previously published article by the same authors. The first article, “Laparoscopy in the management of pediatric vesicoureteral reflux,” by Atul A. Thakre, B. Sreedhar, and C. K. Yeung was published in Indian J Urol. 2007 Oct–Dec; 23(4): 414–419, while the second article, “Technique of Intravesical Laparoscopy for Ureteric Reimplantation to Treat VUR” was published in Advances in Urology in June 2008.
